# Exome sequencing identifies rare mutations of *LDLR* and *QTRT1* conferring risk for early-onset coronary artery disease in Chinese

**DOI:** 10.1093/nsr/nwac102

**Published:** 2022-05-31

**Authors:** Kang Yao, Yuxiang Dai, Juan Shen, Yi Wang, Huanjie Yang, Runda Wu, Qijun Liao, Hongyi Wu, Xiaodong Fang, Shalaimaiti Shali, Lili Xu, Meng Hao, Chenhao Lin, Zhonghan Sun, Yilian Liu, Mengxin Li, Zhen Wang, Qiang Gao, Shuning Zhang, Chenguang Li, Wei Gao, Lei Ge, Yunzeng Zou, Aijun Sun, Juying Qian, Li Jin, Shangyu Hong, Yan Zheng, Junbo Ge

**Affiliations:** Department of Cardiology, Zhongshan Hospital, Fudan University, China; Shanghai Institute of Cardiovascular Disease, China; Department of Cardiology, Zhongshan Hospital, Fudan University, China; Shanghai Institute of Cardiovascular Disease, China; BGI-Shenzhen, China; State Key Laboratory of Genetic Engineering, School of Life Sciences and Human Phenome Institute, Fudan University, China; BGI Genomics, BGI-Shenzhen, China; Department of Cardiology, Zhongshan Hospital, Fudan University, China; Shanghai Institute of Cardiovascular Disease, China; BGI-Shenzhen, China; Department of Cardiology, Zhongshan Hospital, Fudan University, China; Shanghai Institute of Cardiovascular Disease, China; BGI-Shenzhen, China; Department of Cardiology, Zhongshan Hospital, Fudan University, China; Shanghai Institute of Cardiovascular Disease, China; Department of Cardiology, Zhongshan Hospital, Fudan University, China; Shanghai Institute of Cardiovascular Disease, China; State Key Laboratory of Genetic Engineering, School of Life Sciences and Human Phenome Institute, Fudan University, China; State Key Laboratory of Genetic Engineering, School of Life Sciences and Human Phenome Institute, Fudan University, China; State Key Laboratory of Genetic Engineering, School of Life Sciences and Human Phenome Institute, Fudan University, China; State Key Laboratory of Genetic Engineering, School of Life Sciences and Human Phenome Institute, Fudan University, China; State Key Laboratory of Genetic Engineering, School of Life Sciences and Human Phenome Institute, Fudan University, China; Department of Cardiology, Zhongshan Hospital, Fudan University, China; Shanghai Institute of Cardiovascular Disease, China; BGI Genomics, BGI-Shenzhen, China; Department of Cardiology, Zhongshan Hospital, Fudan University, China; Shanghai Institute of Cardiovascular Disease, China; Department of Cardiology, Zhongshan Hospital, Fudan University, China; Shanghai Institute of Cardiovascular Disease, China; Department of Cardiology, Zhongshan Hospital, Fudan University, China; Shanghai Institute of Cardiovascular Disease, China; Department of Cardiology, Zhongshan Hospital, Fudan University, China; Shanghai Institute of Cardiovascular Disease, China; Department of Cardiology, Zhongshan Hospital, Fudan University, China; Shanghai Institute of Cardiovascular Disease, China; Department of Cardiology, Zhongshan Hospital, Fudan University, China; Shanghai Institute of Cardiovascular Disease, China; Department of Cardiology, Zhongshan Hospital, Fudan University, China; Shanghai Institute of Cardiovascular Disease, China; State Key Laboratory of Genetic Engineering, School of Life Sciences and Human Phenome Institute, Fudan University, China; State Key Laboratory of Genetic Engineering, School of Life Sciences and Human Phenome Institute, Fudan University, China; Department of Cardiology, Zhongshan Hospital, Fudan University, China; State Key Laboratory of Genetic Engineering, School of Life Sciences and Human Phenome Institute, Fudan University, China; Department of Cardiology, Zhongshan Hospital, Fudan University, China; Shanghai Institute of Cardiovascular Disease, China

Despite the advances made over the past decades, the known loci for coronary artery disease (CAD) still only explain <20% of the genetic variation in risk [1,2]. The known loci with the strongest effects usually confer a 20%–37% increased CAD risk and the most loci modulate risk by ≤10% [1]. While most of the current data are from European populations, the use of trans-ethnic analyses could be helpful to identify additional loci. Genetic inheritance may impose a high burden on early-onset coronary artery disease (EOCAD). In addition, most of the CAD genetic studies have been conducted in early-onset myocardial infarction [3,4], whose underlying pathophysiological mechanisms might be variable [5]. The gold standard involving angiography for the diagnosis of coronary atherosclerosis would minimize the etiological heterogeneity and enhance the statistical power in the genetic study of EOCAD.

Here, we conducted a prospective, multicenter, coronary angiography-based GRAND study (Genetics and clinical characteristics of coRonary Artery disease in the ChiNese young aDults, GRAND) [6] to explore the genetic predisposition to EOCAD among Han Chinese (Supplementary Fig. S1A). We performed a two-stage analysis using whole-exome sequencing data from 7671 Han Chinese individuals to identify causal mutations in protein-coding regions and susceptible genes for the EOCAD risk (Supplementary Fig. S1). The clinical information of the GRAND study population, including 1950 EOCAD patients with a young age at onset (≤45 years) and 1006 non-CAD older controls (≥65 years, angiographically confirmed), is listed in Supplementary Table S1. In the discovery stage of 1000 randomly selected EOCAD patients and the 1006 non-CAD older controls from GRAND (≥65 years, angiographically confirmed), 1420 common mutations (minor allele frequency (MAF) ≥ 1%, *P* < 0.005), 107 rare mutations (MAF < 1%, odds ratio (OR) > 3.5 and *P* < 0.01) and 85 genes enriched with rare mutations (OR > 3.5 and *P* < 0.01) were selected for replication (Supplementary Fig. S1B). We selected and matched the general controls (20–60 years) from two independent populations [7,8] (Supplementary Methods) to the rest of the EOCAD patients from GRAND based on Euclidean distance in the space of the top 20 principal components to control population stratification (Supplementary Fig. S2 and Supplementary Data). The inflation factor analyses confirmed that the patients and the matched controls had similar genetic backgrounds (Supplementary Fig. S3). Eventually, from the analysis among the 950 EOCAD patients and 4715 genetically matched controls (Supplementary Fig. S2), 27 independent known common variants (*P *< 0.005), three rare mutations and two genes (OR > 3.5 and *P *< 0.05) reached suggestive significance (Supplementary Fig. S1B). When we further combined the populations from the discovery and replication stages with 10 588 Chinese general controls from the China-Map project [9], all of these three rare mutations (two novel mutations in *LDLR*, rs879255066 encoding p.A627T, OR = 117.49; rs875989921 encoding p.W483X, OR = 117.49; both *P* < 6.5 × 10^–7^; one mutation in the novel gene *QTRT1*, rs1195458367 encoding p.R220X, OR = 41.91, *P *< 6.5 × 10^–7^) and two genes (*QTRT1* and *LDLR*, *P *< 2.97 × 10^–6^) achieved exome-wide significance (Fig. 1B and C, Supplementary Fig. S1B and Supplementary Table S2). Sanger sequencing confirmed these three rare mutations at a 100% verification rate.

**Figure 1.**
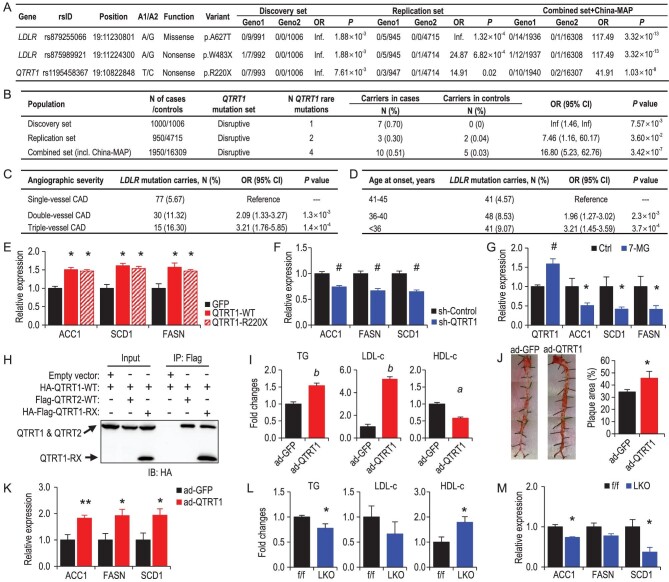
Exome sequencing identifies rare mutations of *LDLR* and *QTRT1* conferring risk for early-onset coronary artery disease. (A) Rare mutations in *LDLR* and *QTRT1* were associated with EOCAD with a significance of *P* < 6.50 × 10^–7^ from two-sided Fisher's exact test. A1, minor allele; A2, major allele; Geno1, the genotype count for the homozygous minor/heterozygous/homozygous major alleles in EOCAD patients; Geno2, the genotype count for the homozygous minor/heterozygous/homozygous major alleles in controls. (B) *QTRT1* gene enriched with rare mutations was associated with EOCAD from gene-wide association analysis with a significance of *P* < 2.97 × 10^–6^ from two-sided Fisher's exact test. (C) Associations between *LDLR* mutations and angiographic severity in EOCAD patients. OR, odds ratio; 95% CI, 95% confidence intervals. ORs were calculated from logistic regression models with adjustment of the top five principal components of ancestry. (D) Associations between *LDLR* mutations and age at onset in EOCAD patients. ORs were calculated from logistic regression models with adjustment of the top five principal components of ancestry. (E) Relative expression of genes related to *de novo* lipogenesis (DNL) in *QTRT1*-overexpressing HepG2 cells with overexpression of wild-type *QTRT1* (*QTRT1-WT*) or mutated *QTRT1* (*QTRT1-R220X*) or GFP control (*GFP*). (F) Relative expression of genes related to DNL in *QTRT1*-knock-down HepG2 cells (sh-*QTRT1*) and control HepG2 cells (sh-Control). (G) Relative expression of *QTRT1* and genes related to DNL in QTRT1 inhibitor (7-methylguanine, 7-MG) treated HepG2. (H) The physical interaction between QTRT1 with QTRT2 and mutated QTRT1. (I) Levels of serum triglycerides (TG), LDL-c and HDL-c in ad-*QTRT1* and ad-GFP mice; *n* = 6–9. (J) Representative images of Oil Red O-stained arteries in ad-*QTRT1* and ad-GFP mice, and quantification of the percentage of plaque area in the Oil Red O-stained arteries; *n* = 6–9. (K) Relative hepatic expression of genes related to DNL in ad-*QTRT1* and ad-GFP control mice; *n* = 6–9. (L) Levels of hepatic triglycerides (TG), LDL-c and HDL-c in liver-specific *QTRT1* knockout mice (LKO) and control flox/flox littermates (f/f); *n* = 5. (M) Relative hepatic expression of genes related to DNL in liver-specific *QTRT1* knockout mice (LKO) and control flox/flox littermates (f/f); *n* = 5. ^*^P < 0.05; ^**^P < 0.01; ^#^*P* < 0.005; *^a^P* < 0.001; *^b^P* < 0.0005. Mann-Whitney test was used for the statistical analyses in Fig. 1E–M.

The two novel rare mutations in *LDLR* from the Chinese population were found to be monomorphic (p.A627T) or absent (p.W483X) in European participants in the Genome Aggregation Database (gnomAD) [10]. Notably, rare *LDLR* copy number variations (Supplementary Fig. S4) were more likely to be found in this study compared with a previous report (nine carriers in 1950 Han Chinese patients with whole-exome sequencing data versus one carrier in 2081 European patients with whole-genome sequencing data) [4]. In our GRAND study population, *LDLR* mutation carriers showed geographical clustering characteristics, mainly from East and Central China (Supplementary Fig. S4). Among EOCAD patients, rare *LDLR* mutations were associated with more severe atherosclerosis (OR [95% confidence interval] of double-vessel disease 2.09 [1.33–3.27], *P* = 0.001; and of triple-vessel disease 3.21 [1.61–3.52], *P* = 1.4 × 10^–4^; Fig. 1C), an earlier onset age (for those with an onset age of 36–40 years 1.96 [1.27–3.02], *P* = 0.002; for those with an onset age of <36 years 3.21 [1.45–3.59], *P* = 3.7 × 10^–4^; Fig. 1D) and a higher risk of major adverse cardiac events in 20 months (adjusted hazard ratio [95% confidence interval] 6.18 [3.86–9.89] in carriers vs. non-carriers, *P* = 3.1 × 10^–14^) (Supplementary Fig. S4 and Supplementary Table S3).

Our study is the first to report that *QTRT1* was associated with CAD risk to the best of our knowledge. We found three extremely rare loss-of-function mutations of *QTRT1* in the China-MAP database (Supplementary Table S2) but not in another large database (PGG.SNV) [11], implying that this gene might be relatively conserved across populations. In the association of *QTRT1* with EOCAD, an extremely rare nonsense mutation (rs1195458367 encoding p.R220X) was the leading mutation. This rare mutation in *QTRT1* had a MAF of 0.256% in patients, 0.0087% in our controls, 0.005% in 10 588 Han Chinese [9] (Supplementary Table S2) and 0.003% in Europeans in gnomAD. EOCAD patients carrying the *QTRT1* p.R220X mutation had higher plasma low-density lipoprotein cholesterol (LDL-c) levels (mean ± SD: 198.26 ± 76.29 mg/dL in carriers and 120.63 ± 65.56 mg/dL in non-carriers, *P* = 0.003) and lower high-density lipoprotein cholesterol (HDL-c) levels (28.67 ± 15.36 mg/dL in carriers and 37.36 ± 12.10 mg/dL in non-carriers, *P* = 0.04). In additional data sets (https://t2d.hugeamp.org/), another intronic *QTRT1* mutation (rs4425006, an extremely rare mutation in East Asians but common in Europeans) was also associated with multiple CAD-related phenotypes (Supplementary Fig. S5).

Because *LDLR* is known to cause hypercholesterolemia and CAD risk [3] and *QTRT1* is the primary novel finding from the above analyses, we employed *in vitro* and *in vivo* experiments to explore the function of *QTRT1*. First, we overexpressed *QTRT1-*p.R220X or wild-type *QTRT1* in human hepatocytes and tested whether it would impact the hepatic lipid metabolism (Supplementary Fig. S6A). Our results showed that both *QTRT1-*p.R220X and wild-type *QTRT1* increased the expression of the essential genes of *de novo* lipogenesis (DNL) (*ACC1*, *FASN* and *SCD1*) (Fig. 1E), suggesting that *QTRT1-*p.R220X might be a gain-of-function mutation and upregulate lipid synthesis. Consistently, the knock-down of *QTRT1* significantly downregulated the expression of the genes related to DNL (Fig. 1F and Supplementary Fig. S6B). In addition, treatment with 7-methylguanine, a known QTRT1 inhibitor, significantly downregulated the expression of *ACC1*, *SCD1* and *FASN* in hepatocytes (Fig. 1G). We noticed a simultaneous increase in the expression of *QTRT1* in the inhibitor-treated cells, suggesting a feedback regulation on *QTRT1* expression (Fig. 1G). Previously, it has been reported that QTRT2 shared a high degree of homology with QTRT1, but the different amino acids in the catalytic area caused the inability of QTRT2 to catalyse the reaction. However, forming a heterodimer with QTRT2 is critical for QTRT1 activity [12]. Thus, we hypothesized that the mutated QTRT1 might also form a protein complex with wild-type QTRT1. To test this hypothesis, we performed a Co-immunoprecipitation experiment between mutated and wild-type QTRT1. Our data confirmed that, similarly to QTRT2, the mutated QTRT1 could physically interact with wild-type QTRT1 (Fig. 1H), which may explain the gain-of-function of the mutated QTRT1. Combined with the above-mentioned *in vitro* data, our observations suggested that QTRT1 regulates DNL in hepatocytes in a cell-autonomous manner.

Next, we tested whether the QTRT1 would play a role in lipid metabolism *in vivo*. We overexpressed *QTRT1* in the liver of *ApoE* null mice through tail vein injection of adenovirus. qPCR data confirmed the expression of *QTRT1* in the livers of overexpressed mice (ad-*QTRT1*) was 2.77-fold higher than that in the control mice (ad-GFP) 3 months after the virus injection (Supplementary Fig. S6C). Furthermore, ad-*QTRT1*-treated mice had significantly higher circulating levels of LDL-c (5.19-fold) and triglycerides (1.54-fold) but lower levels of HDL-c (0.58-fold) compared with the control group (Fig. 1I). A similar but milder trend appeared 2 months after the virus injection (Supplementary Figs. S6D and E), but no difference was observed at 1 month since injection (Supplementary Fig. S6E). *QTRT1* overexpression seemed to result in more arterial plaques stained by Oil Red O (Fig. 1J), which was in line with the higher serum levels of LDL-c and triglycerides in ad-*QTRT1* mice. Consistently with our *in vitro* data, the expression level of essential genes (*ACC1*, *FASN* and *SCD1*) for DNL was upregulated in *QTRT1*-overexpressing mice (Fig. 1K). *QTRT1* overexpression did not affect the expression of other genes related to lipoprotein transportation in the liver (i.e. *LDLR*, *ApoA1* and *ApoB*; Supplementary Fig. S6F). Further, we generated a conditional liver-specific *QTRT1* knockout mouse model to validate the function of *QTRT1* in lipid metabolism (Supplementary Fig. S6G). In these *QTRT1* knockout mice, we observed lower total triglyceride (0.77-fold) and trending lower LDL-c (0.66-fold) but significantly higher HDL-c content (1.79-fold) in the livers (Fig. 1L), which is in line with the observation found in *QTRT1*-overexpressing mice. Consistently, a reduced expression of *ACC1* and *SCD1*, and a trending lower expression of *FASN* were also found in liver-specific *QTRT1* knockout mice compared to wild-type control mice (Fig. 1M). These results confirmed that *QTRT1* was essential in lipid metabolism and atherosclerosis development.

In this most extensive whole-exome sequencing study of CAD among the non-European population, we identified that novel gene *QTRT1* and rare novel mutations in *LDLR* contribute to EOCAD risk. *QTRT1* mutations influenced EOCAD risk through DNL dysregulation and accelerated atherosclerosis. These findings might provide new insights into genetic screening, early diagnosis and future drug discovery for CAD in young individuals.

## Supplementary Material

nwac102_Supplemental_files
