# Supplementary material for: Exome sequencing identifies rare mutations of LDLR and QTRT1 conferring risk for early-onset coronary artery disease in Chinese
Source: Natl Sci Rev. 2022 May 31;9(8):nwac102. doi: 10.1093/nsr/nwac102 (PMC9429139; doi:10.1093/nsr/nwac102)
Supplement: nwac102_Supplemental_files [file nwac102_supplemental_files.zip › Supplementary_Data.docx]

Supplementary Data

Exome sequencing identifies rare mutations of *LDLR* and *QTRT1* conferring risk for early-onset coronary artery disease in Chinese

Kang Yao, Yuxiang Dai, Juan Shen, Yi Wang, Huanjie Yang, Runda Wu, Qijun Liao, Hongyi Wu, Xiaodong Fang, Shalaimaiti Shali, Lili Xu, Meng Hao, Chenhao Lin, Zhonghan Sun; Yilian Liu, Mengxin Li, Zhen Wang, Qiang Gao, Shuning Zhang, Chenguang Li, Wei Gao, Lei Ge, Yunzeng Zou, Aijun Sun, Juying Qian, Li Jin, Shangyu Hong*, Yan Zheng*, and Junbo Ge*

***Corresponding authors (Emails):**

[shangyu_hong@fudan.edu.cn](mailto:shangyu_hong@fudan.edu.cn);

yan_zheng@fudan.edu.cn;

ge.junbo@zs-hospital.sh.cn;

**This PDF document contains:**

Supplementary Methods

Supplementary Results

Supplementary Figures S1-S11

Supplementary Tables S1-S11

Supplementary References 1-18

**Supplementary Methods**

**Study population**

The GRAND study is a prospective, multicenter, hospital-based study. All participants completed a baseline questionnaire about demographic factors, lifestyle, medical history, and medication use at recruitment from May 2017 to May 2018. The detailed information of this study has been described in our previous report (1). In the current analysis of the GRAND population, we included patients with EOCAD (age ≥ 18 and ≤ 45 years) and CAD-free older controls (age ≥ 65 years), and both patients and controls were confirmed by angiography. Moreover, the EOCAD patients were actively followed up for prognosis (e.g., major adverse cardiovascular events [MACEs]) until Dec 31, 2019, in the current analysis (Supplementary Fig. S1).

In this study, CAD was defined by the criteria of at least 50% stenosis of one or more main coronary arteries by coronary angiography. The older controls were men and women older than 65 years and free of CAD (i.e., <30% stenosis by coronary angiography). The general controls from two Han-Chinese cohorts were referred to the general participants aged 20–60 years who were free of self-reported CAD (Supplementary Fig. S1).

The study protocol was approved by the central ethics committee at Zhongshan Hospital, Fudan University, and by institutional review boards and ethics committees at each participating site. An independent data monitoring board has reviewed the data at regular intervals. Written informed consent was received from each participant prior to participation. All procedures performed in studies involving human participants were following the ethical standards of the institutional and/or national research committee and with the 1964 Helsinki declaration and its later amendments or comparable ethical standards.

**Whole-exome (genome) sequencing**

DNA extraction, purification, fragmentation, hybridization, library construction, and sequencing of the GRAND participants were performed at the BGI, as previously described (2). Briefly, the qualified genomics DNA sample was randomly fragmented by Covaris technology, and library fragments of 150–250 bp were obtained. DNA fragments were then hybridized to the exome array (Agilent V5 kit, Agilent Technologies, Santa Clara, CA, USA) for enrichment. The captured libraries were loaded on BGISEQ sequencing platforms to perform high-throughput sequencing at BGI. Finally, a mean coverage of approximately 133.5× with paired-end 100 bp reads were achieved (Supplementary Table S4).

The general controls from the Shenzhen physical examination project (3) were sequenced using a BGISEQ-500 platform at BGI with whole-genome sequencing (WGS) strategy (BGI, Beijing, China), and a mean coverage of 42.74× was achieved (Supplementary Table S4). The general controls from the HUABIAO project (4) were sequenced using an Illumina NovaSeq platform at Beijing iGeneTech with the AlwholeExome array (iGeneTech, Beijing, China), and a mean coverage of 102.81× was achieved (Supplementary Table S4). DNA libraries were constructed following the manufacturer’s protocols.

**Alignment and Variant Calling**

For all the samples from the discovery and replication stages, raw reads were filtered by removing adapters and low-quality sequences. The remaining clean reads of each individual were aligned to reference genome build GRCh37 using Burrows-Wheeler Aligner (BWA) software (0.75)(5). Duplicated reads were marked by Picard (<https://broadinstitute.github.io/picard/>, 2.10.10). Local realignment around indels and base quality score recalibration was performed on BAM files using the Genome Analysis Toolkit (GATK 3.2.2). GATK HaplotypeCaller was used to detect (6) SNVs and small indels according to the best practices recommended by GATK.

**Variants and sample quality control**

In the discovery stage, we applied a series of quality control steps and excluded variants with a minor allele average depth <4, an average depth <8, a low mapping quality score, strand bias or allelic imbalance, or deviations from Hardy–Weinberg equilibrium (Supplementary Table S5A). In total, 21 samples were excluded in the discovery stage, because they either had an average sequencing depth <30, <90% of the target region covered by at least 10×, reads mapping rate <95%, occurred as an outlier of GC content, a 3^rd^ degree or closer relationship with other samples, a population outlier (the EIGENSTRAT 4.2 software(7)), or an abnormal coverage rate of the Y chromosome (Supplementary Table S5B).

In the replication stage, we applied the inclusion criteria as mentioned above (Supplementary Table S5C). Further, we required the candidate SNVs with a read depth of >20X or the candidate genes with 20X coverage for at least 90% of samples (Supplementary Fig. S7).

**Sanger validation for candidate SNVs**

To minimize the possible false-positive rate of exon sequencing, we used Sanger sequencing to verify the presence of candidate variations in *LDLR and QTRT1* from WES analysis. We selected 54 samples for Sanger validation using capillary electrophoresis with an ABI3730xL Genetic Analyzer (Applied Biosystems, Foster City, CA, USA) in the BGI sequencing center. Using deep sequencing, we obtained a 100% verification rate and presented the result for *QTRT1* in Supplementary Fig. S8.

**CNV detection, quality control, and validation by real-time PCR**

XHMM software(8) (1.0) and CoNIFER pipeline(9) (0.2.2) were used in parallel to discover rare CNVs at a single-exon resolution in the discovery and replication stages, respectively. Finally, rigorous quality control yielded ~800 CNVs at a frequency level of <0.01 for all subsequent analyses (Supplementary Table S6A). Nine rare CNVs in *LDLR* from the nine EOCAD patients were validated by real-time quantitative PCR (qPCR)(10) using the SYBR Green system (Takara Biomedical Technology, Beijing, China) on a StepOnePlus Real-Time PCR System (Applied Biosystems, Singapore). The thermal cycling conditions were as follows: one cycle of 95 °C for 5 min followed by 40 cycles of denaturation at 95 °C for 10 s and annealing/extension at 60 °C for 30 s. The primers for CNV analysis were designed by the Primer-BLAST web tool **(**Supplementary Table S6B**)**, where *GAPDH* and *HPRT1* genes were treated as the standard internal references for genomic qPCR (10). The qPCR primers were also checked by analyzing the melting curves obtained during the qPCR assay. The experiments for two healthy controls and five EOCAD patients were run in triplicate. The mean values and standard deviations of the data were used for statistical analyses using the comparative ΔΔ Ct cycle threshold method.

**Case-control matching using principal component analysis**

Population structure, which can lead to spurious correlations between allele frequencies and non-genetic risk factors, has long been a significant confounding factor for genetic association studies. Testing and correcting for population structure is a standard practice through genomic inflation factors and principal component (PC) analysis methods.

In the replication stage, the initial participants included 984 EOCAD patients from the GRAND study and 7057 general controls from two external populations (3, 4) (Supplementary Fig. S1), i.e., 2057 WGS (average depth ~42.7×) samples from the Shenzhen healthy physical examination study (3) and 5000 WES (average depth ~102.8×) samples from HUABIAO project (4) (https://www.biosino.org/wepd/, Supplementary Fig. S1, and Supplementary Table S4). Euclidean distance in the top 20 principal component space was used to match controls to EOCAD patients. In brief, we calculated the Euclidean distance of each control to all EOCAD patients. We got the density distribution of Euclidean distance across all participants and defined different outlier criteria of Euclidean distance to search for the best-matched controls (Supplementary Fig. S2A). We stopped searching until the t-statistic of the principal components between cases and controls was not significant (Supplementary Table S7) and used the genomic inflation factors to assess the genetic matching effect (Supplementary Fig. S3D). Finally, 4715 general controls clustered closely with 950 patients and were included in the replication stage (Fig. 1). Based on these participants, we carried out the GATK genotype joint calling for candidate single-nucleotide variants (SNVs) and genes selected from the discovery stage.

**SNV association analysis for EOCAD**

An additive genetic model using logistic regression adjusted for the top five principal components for the common single-nucleotide variants (SNVs, minor allele frequency (MAF) >1%) and Fisher’s exact test for rare SNVs (MAF < 1%). We performed association analysis using PLINK (version 1.90)(8), separately in the discovery and replication stages. We used a significance threshold of P < 6.50×10^−7^ (Bonferroni correction for 47,567 common variants using linkage disequilibrium pruning with the parameter --maf 0.01 --indep-pairwise 100 5 0.1 in Plink) and 29,309 rare protein-altering SNVs (MAF<1% and at least three mutations in EOCAD patients).

For common variants, we presented the association result combining the discovery and replication stages as the primary results. For the rare variants and genes, we further integrated 10,588 Chinese general controls from China-Map project (https://mbiobank.com)(11) into the samples from the discovery and replication stages to improve the statistical power. We presented the resulting signals as the primary results for rare variants and genes.

**Gene-based association analysis for EOCAD**

Five protein prediction algorithms from ANNOVAR(12) (2017) (LRT score, MutationTaster, PolyPhen-2 HumDiv, PolyPhen-2 HumVar, and SIFT) were applied to nonsynonymous SNVs to identify the most possible rare damaging SNVs (MAF < 1%). Then, three SNV sets were defined, including 1) a “deleterious (broad)” set comprising missense mutations annotated as “deleterious” by at least one algorithm, nonsense, splice-site, and indel frameshift mutations; 2) a “deleterious (strict)” set comprising missense mutations annotated as “deleterious” by all five protein prediction algorithms, nonsense, splice-site, and indel frameshift mutations; 3) “disruptive” mutations only (nonsense, splice-site, or indel frameshift). Then collapse (13) (two-tailed Fisher’s exact test by R 3.6.1 software) and SKAT (14) (EPACTS 3.2.6 software, adjusted for the top five PCs) were used to perform gene-wide association analysis on three defined SNV sets in the discovery stage (Supplementary Fig. S9). For gene-wide tests, we used a significance threshold of P < 2.97×10^-6^, corresponding to a Bonferroni correction for 16,790 tested genes.

**Variance explained by candidate variants**

A method proposed by *So* and colleagues was used to estimate the variance explained in the present study (15). CAD prevalence in calculations was set to 5%, according to a previous study (13).

**Ascertainment of *LDLR* familial hypercholesterolemia mutations**

Based on previously reported principles (13, 16) for the identification of familial hypercholesterolemia mutations, *LDLR* rare variations (MAF<1%) meeting any of the following conditions were considered as pathological: 1) missenses mutations that were annotated as “deleterious” by all five protein prediction algorithms from ANNOVAR (12) (LRT score, MutationTaster, PolyPhen-2 HumDiv, PolyPhen-2 HumVar, and SIFT); 2) nonsense, splice-site or indel frameshift variants; 3) variants annotated as “Pathogenic” or “Likely pathogenic” by the Clivar database (17).

**Clinical phenotype analysis**

The circulating levels of total cholesterol and low-density lipoprotein cholesterol (LDL-c) for participants using lipid-lowering medications were adjusted by calculating total cholesterol/0.8 and LDL-C/0.7 (18). A two-sided Wilcoxon test was used to calculate the statistically significant level for candidate mutations and genes. The impact of aggregations of *LDLR* rare mutations on atherosclerotic coronary burden and disease onset age among EOCAD patients was assessed using logistic regression in R software (3.6.1) with adjustment of the first five principal components of ancestry.

**Survival analysis**

Survival analysis was conducted using R software (3.6.1, survival package). The hazard ratio and P-value of developing MACEs for the *LDLR* rare mutation carriers were calculated from Cox proportional hazard regressions, with adjustment of age, sex, body mass index (BMI), smoking status (ever vs. never smokers), diabetes status, systolic blood pressure, high-density lipoprotein cholesterol (HDL-c), total cholesterol, and the first five principal components of ancestry.

**Functional validation in mice model**

Eight-week-old ApoE null mice (GemPharmatech Co., T001458) were fed a high-fat and high-cholesterol diet (Research Diets Inc., D12108c). All experiments were performed following the ethical guidelines of Fudan University. The triglyceride (TG), HDL-C, and LDL-C levels in mouse serum were measured using kits from Nanjing Jiancheng Bioengineering Institute (A110-1-1, A112-1-1, A113-1-1) following the manufacturer’s instructions.

**Coimmunoprecipitation** 48h after transfecting of HA -QTRT1-WT in 293T cells coexpressing HA-FLAG-QTRT1-R220X, or pENTER as vector control, or FLAG-QTRT2-WT as a positive control, the cells were rinsed with PBS and harvested in cell lysis buffer for Western and IP (Beyotime, P0013) added with 1× Protease inhibitor cocktail (Beyotime, P1006) and UltraNuclease (Yeasen, 20156ES25). Total protein was isolated by centrifugation at 12,000 rpm× 30 min at 4 ℃ and partly preserved as input, while the rest of the supernatant was treated with High Capacity Neutravidin Agarose (Thermofisher, 29202) and then incubated with anti-FLAG M2 affinity gel (Sigma, F2426) overnight. The coimmunoprecipitated protein was eluted with 0.1 M glycine HCl at pH 3.5 and loaded for immunoblotting.

**Cell culture and treatment**

Single-nucleotide mutations in *QTRT1* (R220X) were generated by the QuikChange Multi Site-Directed Mutagenesis Kit (Stratagene, San Diego, CA, USA; #200514 and #200515). Adenovirus were purchased from WZ Biosciences Inc. Human HepG2 hepatocytes (ATCC, HB-8065) were cultured in low-glucose Dulbecco’s modified Eagle’s medium with 10% fetal bovine serum. HepG2 cells were infected with adenovirus containing shRNA targeting *QTRT1* or adenovirus to overexpress wild-type *QTRT1* or *QTRT1* (R220X). Three days after infection, total RNA was harvested for qPCR analysis. Primary hepatocytes were isolated from 8-week-old mice, which was anesthetized and perfused via the post cava with buffer and subsequent digestion solution containing collagenase type I (Sigma, C0130). The digested liver was excised, minced, filtered, and centrifugated, and cells were seeded in plates precoated with collagen type I solution from rat tail (Sigma, C3867). 7-methylguanine (Sigma, 67073) was dissolved in 1M NaOH, and 24 hours after treatment, the total RNA of primary hepatocytes was harvested for qPCR.

**Liver-specific knockout mice model of *Qtrt1***

A conditional knockout mouse model of *Qtrt1* was established by knocking in the *LoxP* cassettes around the fourth to tenth exon (*Qtrt1^flox/flox^*). By crossing the *Qtrt1^flox/flox^* mice to *Alb-cre* mice, we created the hepatocyte-specific *Qtrt1* knockout mouse model *Alb-cre:: Qtrt1^flox/flox^*.

**Mice and Treatment and TG, HDL-C, and LDL-C measurements**

Eight-week-old *ApoE* null mice (GemPharmatech Co., T001458) were fed a high-fat and high-cholesterol diet (Research Diets Inc., New Brunswick, NJ, USA; D12108c). Adenoviruses were administered via tail vein injection. All experiments were performed following the ethical guidelines of Fudan University.

The TG, HDL-C, and LDL-C levels in mouse serum were measured using kits from Nanjing Jiancheng Bioengineering Institute (Nanjing, China; Cat#: A110-1-1, A112-1-1, A113-1-1) following the manufacturer’s instructions.

**Availability of data and materials**

The WES data in the GRAND study have been deposited in the China National GeneBank database under the accession code CNP0000730. Other data supporting the findings of this study are available from the corresponding authors upon reasonable request.

**Supplementary Results**

**Association analysis**

In the discovery stage, our participants had an average of 43 nonsense, 4736 missense, 43 splice-site, and 53 insertion or deletion (indel) mutations (Supplementary Fig. S10). In total, 141,587 common (MAF >5%, 7.57%), 61,396 low-frequency (MAF 1–5%, 3.28%), 1,668,528 rare variants (MAF < 1%, 89.15%) and 16,790 genes (Supplementary Table S11) were included to perform single-variant (Supplementary Fig. S11) and gene-wide association analyses (Supplementary Fig. S9). 1420 common mutations (minor allele frequency (MAF)≥1%, P < 0.005), 107 rare mutations (MAF<1%, odds ratio (OR) > 3.5 and P < 0.01) and 85 genes enriched with rare mutations (OR > 3.5 and P < 0.01) were selected for replication.

In the combined analysis of discovery and replication populations, three independent common SNVs (rs1051338 in *LIPA*, encoding p.T16P; rs2127898 in *ADAMTS7*, encoding p.T307M; rs10965215 in *CDKN2B-AS1*) achieved whole exome-level significance (P<6.5×10^−7^) (Supplementary Fig. S11, Supplementary Table S8A), and they were all in tight linkage with previously reported loci (Supplementary Table S8B), together explaining 1.3% of the EOCAD variance.

***LDLR* rare mutations**

In this study, 87 rare missense mutations (previously annotated as pathogenic in the ClinVar online database or predicted to be damaging by each of 5 computer prediction algorithms, Supplementary Methods and Supplementary Fig. S4), 9 CNVs and 9 small deletions/insertions in *LDLR* were identified as the familial hypercholesterolemia mutations, and 130 EOCAD patients carried these SNVs (6.7% of 1,950 patients). Among the 130 *LDLR* rare-mutation carrier patients (6.7% of 1,950 patients), 8 carried multiple *LDLR* mutations, and their average LDL-C level (412.3 mg/dL) was much higher than that in noncarriers or in single-mutation carriers (Supplementary Fig. S4C, Supplementary Table S9). Under the same definition mutation set, rare mutations of *LDLR* from the current Chinese population seemed to exhibit a larger effect on EOCAD (Supplementary Table S10).

**Supplementary Figures**

**Figure S1. The flowchart of study population selection (A) and genetic analysis (B).** A. The light blue boxes represent the GRAND study, a prospective, multicenter, hospital-based study including 1950 EOCAD patients and 1006 older controls after quality control and genetic match. The gray boxes represent two independent Han-Chinese studies (Methods), which were included as general controls in the replication stage. In the GRAND study, 95.6% of patients with EOCAD were followed up for a median of 20 months to document major adverse cardiovascular events. B. The flowchart of genetic analysis.

**A**

**B**
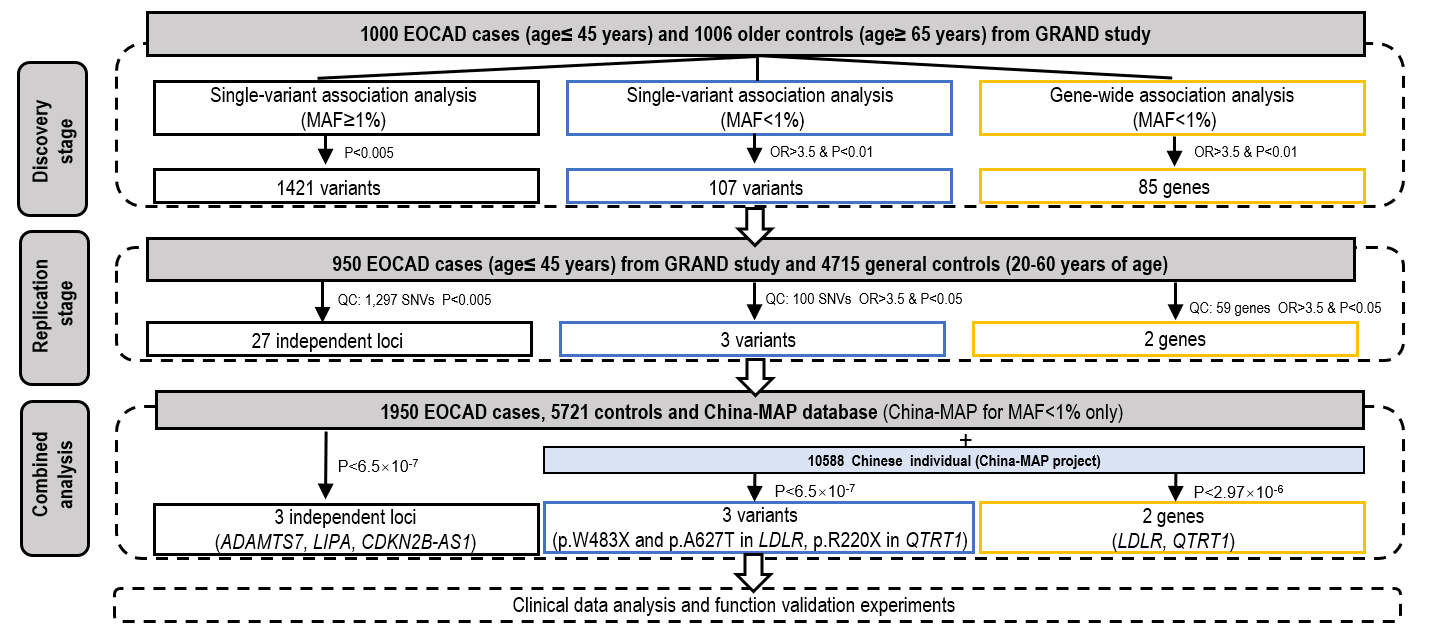


# Figure S2. The genetic matching process between EOCAD patients and general controls. In the replication stage, two additional populations of 2057 WGS and 5000 WES Chinese individuals, were selected to match cases. We used plink (--maf 0.05 --indep-pairwise 100 5 0.1) to generate a pruned subset of SNVs based on the overlapping variants. The principal components (PCs) were calculated using these pruned SNVs (26,301). We calculated the Euclidean distance between the control and all EOCAD patients for each control. We calculated the density distribution of Euclidean distance across all samples and defined different outlier criteria of Euclidean distance to search the matched controls (A). We stopped searching until the T-test of the principal components between cases and controls was not significant. Finally, we obtained 4715 genetic-matched general controls, including 1213 Han Chinese WGS and 3502 Han Chinese WES data (B).

**Figure S3. Principal component analyses and quantile-quantile plots in GRAND population from the discovery stage (A, C) and the replication stage (B, D).** Plots for two PCs among the first to the fourth components were drawn. The corresponding genomic inflation factors were 1.06 in the discovery stage **(C)** and 1.07 in the replication stage **(D)**.

**Figure S4. *LDLR* mutation spectrum and characteristics.** (A) The mutations were depicted according to their genomic position. Each circle represents one sample. The red and cyan circles represent mutations in patients and controls, respectively. (B) Validation of exome-based copy number variant (CNV) calls by real-time PCR. Nine CNVs, including five deletions and four duplications, are shown. (C) The LDL-c levels in different *LDLR* carrier groups. (D) The survival curve of major adverse cardiovascular events (MACEs) according to the *LDLR* mutation status. Among EOCAD patients who were followed up for a median of 20 months (interquartile range, 16–25 months), 143 developed MACEs (including 2 cardiac deaths, 4 nonfatal MIs, and 137 ischemia-driven revascularizations). Hazard ratios were calculated using Cox proportional hazard regressions with adjustment of age, sex, BMI and smoking status, diabetes status, systolic blood pressure, HDL-c, total cholesterol, and the first five principal components of ancestry. (E) Distribution of *LDLR* pathogenic mutation carriers in different provinces in China

**Figure S5. Association signals of *QTRT1* from the T2D Knowledge Portal (https://t2d.hugeamp.org/). (A)** An intronic variant rs4425006 in *QTRT1* was linked to multiple CAD-related phenotypes. **(B)** The frequency distribution of rs4425006 in the 1000 Genomes Project.

***
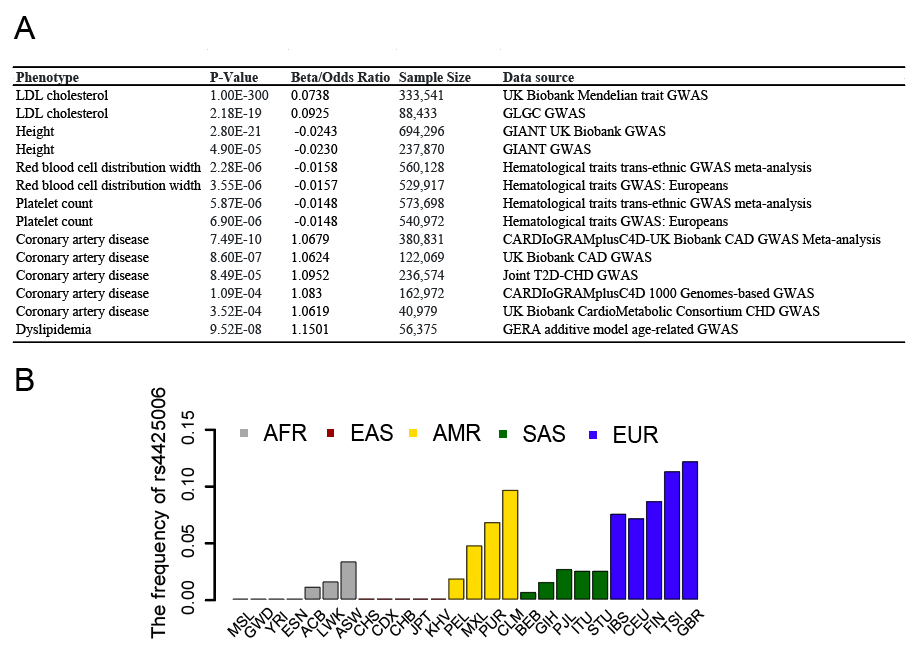
***

**Figure S6. *QTRT1* affected hepatic *de novo* lipogenesis and serum lipid profiles.** (A) Relative expression of *QTRT1* in *QTRT1*-overexpressing HepG2 cells with overexpression of wild-type *QTRT1* (*QTRT1-WT*), or mutated *QTRT1* (*QTRT1-R220X*) or GFP control (*GFP*); (B) Relative expression of *QTRT1* in *QTRT1*-knockdown HepG2 cells (sh-*QTRT1*) and control HepG2 cells (sh-Control); (C) The relative *QTRT1* mRNA levels in the livers of *QTRT1*-overexpressing (ad-*QTRT1*) and control (ad-GFP) mice. *n* = 6–9. (D) Levels of serum LDL-c, TG, and HDL-c in *QTRT1*-overexpressing mice (as-*QTRT1*) and control mice (ad-GFP) two months after virus injection; n=6~9. (E) Levels of serum LDL-c, TG, and HDL-c in *QTRT1*-overexpressing mice (as-*QTRT1*) and control mice (ad-GFP) one month after virus injection; n=6~9. (F) Relative hepatic expression of lipoprotein transporters in ad-*QTRT1* and ad-GFP mice; *n* = 6–9. (G) Relative expression of *Qtrt1* in liver-specific *Qtrt1* knockout mice (LKO) and control flox/flox littermates (f/f); *n* = 5. *, p<0.05; #, p<0.005; ns, not significant. Mann-Whitney test was used for all statistical analyses.


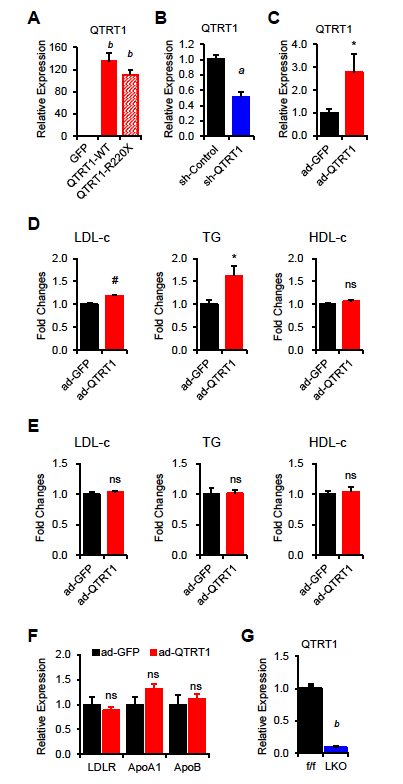


**Figure S7. 20× coverage of samples in the replication stage across candidate mutations and genes selected from the discovery stage.** The number in red color is the number of the analyzed SNVs and genes in the replication stage.

**
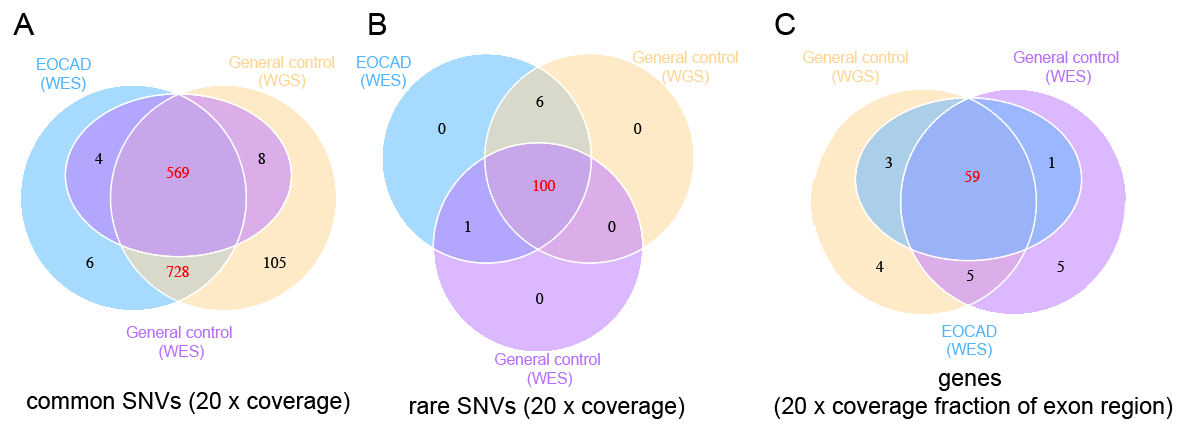
**

**Figure S8. Sanger sequencing of *QTRT1* p.R220X in the discovery stage.** 7 carriers of EOCAD patients and two carriers of controls were selected for validation. We obtained a 100% verification rate.


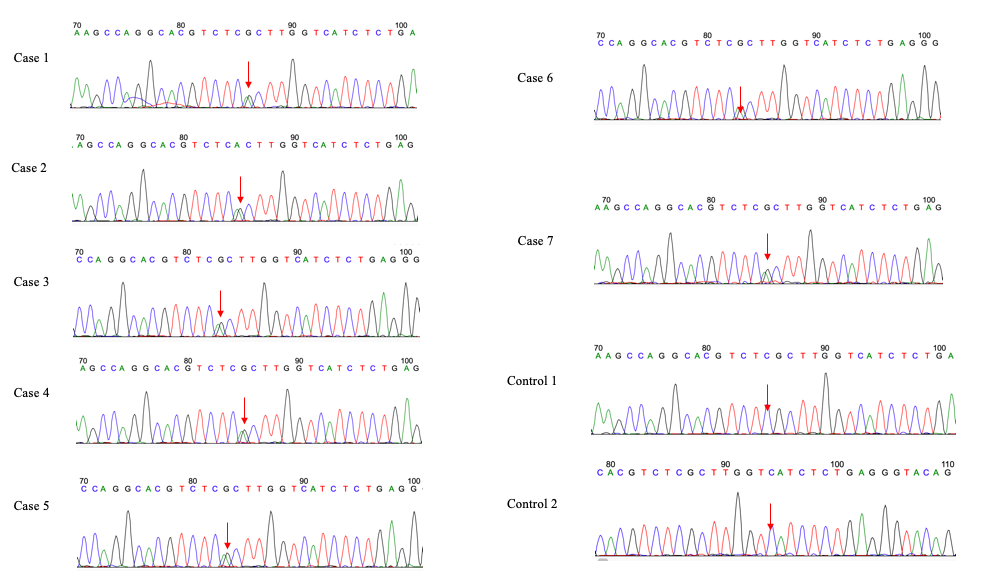


**Figure S9. Quantile-quantile plots of gene-wide association signals in the discovery stage (1000 cases and 1006 older controls).** The collapse **(A)** and SKAT methods **(B)** with adjustments of the top five principal components were employed in gene-wide analysis; only *LDLR* exceeded a gene-wide significance level of P<2.97×10^−6^ (0.05/16790) in the discovery stage.

*
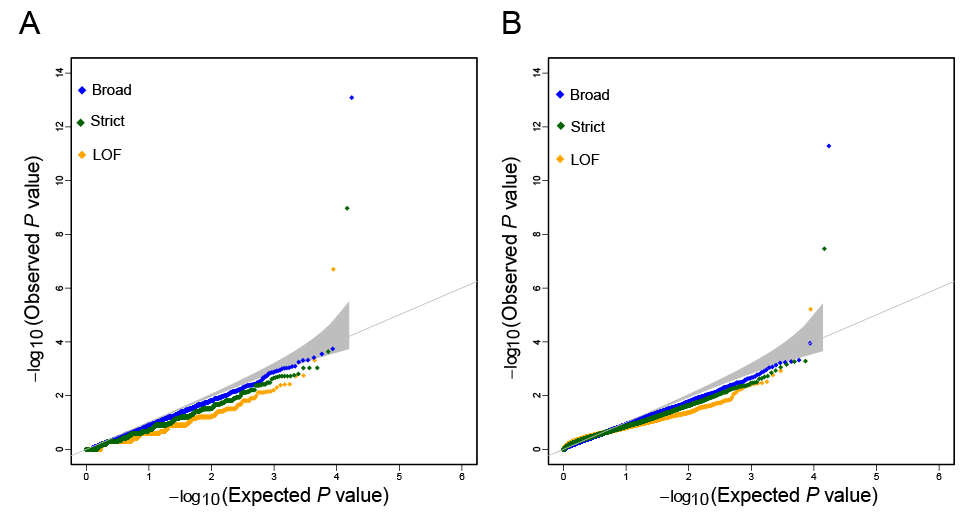
*

**Figure S10. The number of mutations carried by EOCAD patients and controls across different mutation types in the discovery stage.**

***
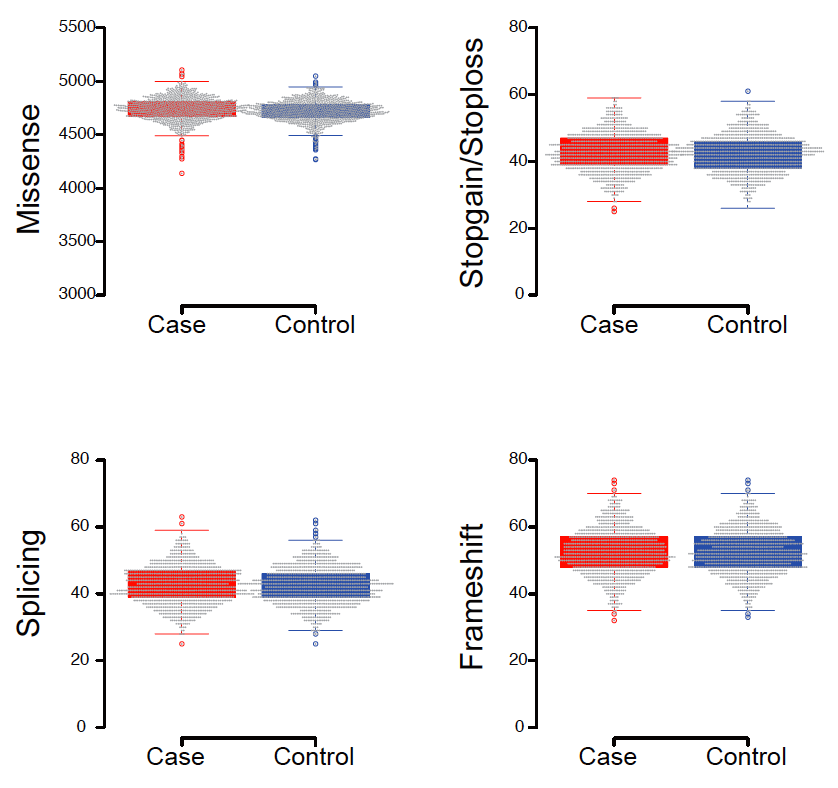
***

**Figure S11. Common variants were associated with EOCAD.** Manhattan plot of common variants in the discovery stage (1000 cases vs. 1006 controls). The red dashed line depicts the threshold of P<6.50×10^−7^. The triangle denotes the functional mutations, including missense, nonsense, frameshift, and splicing mutations. The red diamonds and triangles denote identified SNVs in both two stages with P<0.005 and a combined P<6.50×10^−7^.

**Supplementary Tables** (see supplementary tables in the attached excel file)

**Table S1.** Baseline characteristics of patients with EOCAD and control participants in the discovery and replication stages.

**Table S2.** The association of rare variants and genes with EOCAD among participants from discovery and replication stages and general controls from the China-MAP database.

**Table S3.** Survival analysis of MACEs according to *LDLR* mutation under different models. The hazard ratio was calculated from Cox proportional hazard regressions with adjustment of different covariates and the first five principal components of ancestry.

**Table S4.** Summary of whole exome or genome sequencing across all individuals before quality control.

**Table S5.** Summary of the quality control filters across all individuals in the discovery and replication stages.

**Table S6.** The summary of CNV quality control and qPCR validation.

**Table S7.** The significant T-test of the principal components between EOCAD patients and controls at different genetic matching criteria.

**Table S8.** The association signals of three candidate common mutations and their linkage disequilibrium relationship with previously reported variants.

**Table S9.** Differences in baseline characteristics between carriers of single- and multiple- *LDLR* mutations.

**Table S10.** *LDLR* effect size comparison across different studies under the same definition mutation set.

**Table S11.** Allele frequency spectrum for different mutation sets in the discovery stage.

**Supplementary References**

1. Shalaimaiti, S, Dai, Y, Wu, H*, et al.* Clinical and genetic characteristics of coronary artery disease in Chinese young adults: Rationale and design of the prospective Genetic characteristics of coRonary Artery disease in ChiNese young aDults (GRAND) study. *Cardiol Plus*. 2021; **6**(1): 65-72.

2. Huusko, JM, Karjalainen, MK, Graham, BE*, et al.* Whole exome sequencing reveals HSPA1L as a genetic risk factor for spontaneous preterm birth. *PLoS Genet*. 2018; **14**(7): e1007394.

3. Liu, X, Tong, X, Zou, Y*, et al.* Mendelian randomization analyses support causal relationships between blood metabolites and the gut microbiome. *Nat Genet*. 2022; **54**(1): 52-61.

4. Hao, M, Pu, W, Li, Y*, et al.* The HuaBiao project: whole-exome sequencing of 5000 Han Chinese individuals. *J Genet Genomics*. 2021; **48**(11): 1032-5.

5. Li, H, Durbin, R. Fast and accurate short read alignment with Burrows-Wheeler transform. *Bioinformatics*. 2009; **25**(14): 1754-60.

6. McKenna, A, Hanna, M, Banks, E*, et al.* The Genome Analysis Toolkit: a MapReduce framework for analyzing next-generation DNA sequencing data. *Genome Res*. 2010; **20**(9): 1297-303.

7. Price, AL, Patterson, NJ, Plenge, RM*, et al.* Principal components analysis corrects for stratification in genome-wide association studies. *Nat Genet*. 2006; **38**(8): 904-9.

8. Fromer, M, Purcell, SM. Using XHMM Software to Detect Copy Number Variation in Whole-Exome Sequencing Data. *Curr Protoc Hum Genet*. 2014; **81**: 7 23 1-1.

9. Krumm, N, Sudmant, PH, Ko, A*, et al.* Copy number variation detection and genotyping from exome sequence data. *Genome Res*. 2012; **22**(8): 1525-32.

10. Krepischi, AC, Achatz, MI, Santos, EM*, et al.* Germline DNA copy number variation in familial and early-onset breast cancer. *Breast Cancer Res*. 2012; **14**(1): R24.

11. Cao, Y, Li, L, Xu, M*, et al.* The ChinaMAP analytics of deep whole genome sequences in 10,588 individuals. *Cell Res*. 2020; **30**(9): 717-31.

12. Wang, K, Li, M, Hakonarson, H. ANNOVAR: functional annotation of genetic variants from high-throughput sequencing data. *Nucleic Acids Res*. 2010; **38**(16): e164.

13. Do, R, Stitziel, NO, Won, HH*, et al.* Exome sequencing identifies rare LDLR and APOA5 alleles conferring risk for myocardial infarction. *Nature*. 2015; **518**(7537): 102-6.

14. Lee, S, Emond, MJ, Bamshad, MJ*, et al.* Optimal unified approach for rare-variant association testing with application to small-sample case-control whole-exome sequencing studies. *Am J Hum Genet*. 2012; **91**(2): 224-37.

15. So, HC, Gui, AH, Cherny, SS*, et al.* Evaluating the heritability explained by known susceptibility variants: a survey of ten complex diseases. *Genet Epidemiol*. 2011; **35**(5): 310-7.

16. Khera, AV, Chaffin, M, Zekavat, SM*, et al.* Whole-Genome Sequencing to Characterize Monogenic and Polygenic Contributions in Patients Hospitalized With Early-Onset Myocardial Infarction. *Circulation*. 2019; **139**(13): 1593-602.

17. Landrum, MJ, Lee, JM, Benson, M*, et al.* ClinVar: improving access to variant interpretations and supporting evidence. *Nucleic Acids Res*. 2018; **46**(D1): D1062-D7.

18. Lu, X, Peloso, GM, Liu, DJ*, et al.* Exome chip meta-analysis identifies novel loci and East Asian-specific coding variants that contribute to lipid levels and coronary artery disease. *Nat Genet*. 2017; **49**(12): 1722-30.
